# Maternal and peer attachment, identity formation, and non-suicidal self-injury: a longitudinal mediation study

**DOI:** 10.1186/s13034-019-0267-2

**Published:** 2019-01-19

**Authors:** Amarendra Gandhi, Koen Luyckx, Geert Molenberghs, Imke Baetens, Lien Goossens, Shubhada Maitra, Laurence Claes

**Affiliations:** 1 Faculty of Psychology and Educational Sciences, KU Leuven, Tiensestraat 102, 3000 Leuven, Belgium; 2 UNIBS, University of the Free State, Bloemfontein, South Africa; 3 Interuniversity Institute for Biostatistics and Statistical Bioinformatics, Hasselt University, Hasselt, Belgium; 4 Department of Clinical and Life Span Psychology, Vrije Universiteit Brussels, Brussels, Belgium; 5 Department of Developmental, Personality and Social Psychology, Ghent University, Ghent, Belgium; 6 Center for Health and Mental Health, Tata Institute of Social Sciences, Mumbai, India; 7 Faculty of Medicine and Health Sciences (CAPRI), University of Antwerp, Antwerp, Belgium

**Keywords:** Longitudinal follow-up study, Non-suicidal self-injury, Maternal and peer attachment, Identity synthesis and confusion, Cross-lagged mediation, Parallel process latent growth class mediation models

## Abstract

**Background:**

Non-suicidal self-injury (NSSI) is defined as the repetitive, direct, and deliberate destruction of one’s body tissue without an intention to die. Existing cross-sectional research indicates that the association between maternal/peer attachment and NSSI is mediated by identity synthesis and confusion. However, longitudinal confirmation of the aforementioned mediation models is necessary as cross-sectional models are known to be biased. Consequently, the aim of the present study was to investigate whether identity formation mediates the association between attachment and NSSI in a longitudinal design.

**Methods:**

Three waves of self-report questionnaires data (1 year apart) were collected on maternal and peer attachment, identity, and NSSI from students of a high school in Belgium (at Time 1: Mean age = 15.0 years, SD = 1.85, range = 11–19 years, 50.6% female). Both cross-lagged (between-person) and parallel process latent growth curve (within-person) mediation analyses were used to test the mediation models.

**Results:**

Findings of the cross-lagged analyses indicated unidirectional associations among the study variables, that is, from attachment to identity to NSSI. Parallel process latent growth mediation analyses showed that the association between the slope of maternal attachment and the slope of NSSI was mediated by the slopes of identity synthesis and confusion. Peer attachment models did not fit the data.

**Conclusion:**

The current work demonstrated that dysfunctional maternal and peer attachment may lead to disturbances in identity formation, which, in turn, may lead to increased NSSI. Additionally, within-person analysis indicated that the growth rate of maternal attachment predicted the growth rate of NSSI through the growth rate of identity synthesis and confusion. The clinical relevance of these findings is discussed.

**Electronic supplementary material:**

The online version of this article (10.1186/s13034-019-0267-2) contains supplementary material, which is available to authorized users.

## Background

Non-suicidal self-injury (NSSI) is defined as the repetitive, direct, and deliberate destruction of one’s body tissue without an intention to die, for purposes not socially sanctioned [1]. According to recent reviews and meta-analyses, about 17.2% of adolescents, 13.4% of young adults, and 5.5% of adults have a history of engaging in at least one episode of NSSI in their lifetime, indicating that the risk of NSSI is higher in adolescents than in other age groups [2, 3]. The prevalence of NSSI indeed peaks between 15 and 17 years of age [4]. Disturbances in the process of identity formation—a key developmental task that begins during adolescence—have been identified as an important factor that can increase the risk of NSSI [5]. However, both NSSI and identity formation are also known to be influenced by interpersonal factors like attachment [6, 7]. Given the interrelatedness of these variables, developing integrative models that incorporate attachment and identity development may be necessary to understand how the interplay of these variables can increase the vulnerability to NSSI. Using baseline data from the present study, Gandhi and colleagues [8] tested a mediation model combing these variables and demonstrated that identity synthesis/confusion mediated the association between dimensions of adolescents’ maternal/peer attachment and NSSI. However, longitudinal confirmation of mediation models may be necessary as cross-sectional mediation analysis may often falsely detect indirect effects that cannot be detected by more appropriate longitudinal methods [9]. Therefore, the present study extends the work of Gandhi and colleagues [8] using longitudinal mediation approaches.

### Identity formation and NSSI

The process of identity formation during adolescence begins with a phase of identity crisis—a normative developmental phase of transition in which one’s childhood identity is no longer experienced as suitable, but a new identity is yet to be established [10]. An identity crisis can be resolved in two ways: (a) identity synthesis—a successful resolution of the identity crisis leads to a development of self-identified ideals, values, and goals. Identity synthesis leads to a coherent sense of self which is consistent across time and is often associated with higher self-esteem, purpose in life, and sense of control [11]; (b) identity confusion—if the crisis persists, identity confusion ensues [12]. Identity confusion in adolescents is often associated with an inability to form intimate relationships, mood swings, rebelliousness, and in extreme cases, psychiatric symptoms [13].

There is increasing evidence to suggest that disturbances in identity formation and NSSI may be associated with each other [14–16] Using the data collected at Time 1 (T1) and Time 2 (T2) of the present 2-years follow-up study, Gandhi and colleagues [5] demonstrated that the association between identity synthesis/confusion and NSSI may in fact be bi-directional. This means that lowered identity synthesis and higher identity confusion can lead to increased vulnerability to NSSI. However, engagement in NSSI may also be detrimental to identity synthesis and may increase identity confusion. Although there is increasing evidence supporting the association between disturbances in identity formation and NSSI, the theoretical reasons explaining this association are not clear as of yet. Some researchers have hypothesized that individuals experiencing identity disturbances may experience more negative symptoms secondary to a lack of a consistent sense of self. NSSI may help such individuals to regulate this heightened negative arousal state [5, 16]. Yet, others have suggested that some individuals engaging in NSSI may start using it as a means of developing a sense of identity (“I’m a self-injurer”) which they use to connect with others engaging in similar behaviors [17]. However, more empirical research is needed to confirm these suggested mechanisms.

### Attachment and NSSI

Attachment is a deep and enduring emotional bond that connects one person to another across time and space [18]. Early life experiences with caregivers have a strong influence on the development of attachment in the later years [19]. An infant’s attachment to its caregiver is a source of security when it experiences discomfort or threat. If the caregiver is responsive to and continuously regulates the infant’s emotional states, it develops a secure attachment pattern. An inconsistent response or a lack of response from the caregiver generally leads to disordered forms of attachment [20]. Associations between dysfunctional parent–child attachment and self-harming behaviour have been supported by extensive theoretical and empirical evidence. For example, work of Calkins and Hill [21] and Linehan [22] suggests that caregiver–child interactions inform how infants experience hyper-arousal and de-escalation in arousal. If the caregiver is not responsive to the distress expressed by the infant, the infant may develop an unhealthy working model of affect-related expectations that eventually gets internalized and may generalize to all social interactions. Consequently, individuals with a disturbed sense of attachment may rely on self-destructive strategies like NSSI for affect regulation as a way to compensate for a perceived lack of interpersonal support [23].

The theoretical observations mentioned above have been extensively supported in the existing empirical research [24, 25]. In the extant literature, attachment has been measured in two different forms, that is, from a categorical perspective (i.e., discrete styles of attachment) and a dimensional perspective (i.e., attachment as being represented by multiple underlying processes; [26]). In the present study, we focus only on studies that have used a dimensional approach to study the association between attachment and NSSI, as the dimensional approach has been shown to be more precise and robust in measuring individual differences in attachment [26]. The Inventory of Parental and Peer Attachment (IPPA, [27]) is one of the most frequently used questionnaires in the NSSI literature to measure the communication, trust and alienation dimensions of attachment. Present research has indicated that parental trust and communication are negatively associated with NSSI. On the other hand, more alienation by parents is shown to increase one’s vulnerability to NSSI [28]. Of the three dimensions, alienation may be the strongest predictor of NSSI. This observation is supported by findings of Yurkowski and colleagues [29] who observed that in a large sample of adolescents, higher parental alienation was associated with higher odds of engaging in NSSI. Additionally, these authors also reported that parental alienation increased the probability of engaging in NSSI by increasing emotional dysregulation. In summary, as suggested by previous research [21–23], an invalidating environment (characterized by increased alienation and lack of communication and trust towards parents) seems to be a significant developmental issue that may increase one’s vulnerability to NSSI.

As individuals enter adolescence, relationships with and attachment to peers become increasingly important in the development of adolescents [30]. Although social support from friends has been shown to be protective against the emergence of NSSI [31], the existing evidence does not equivocally support an association between the quality of peer attachment and NSSI. More specifically, some studies indeed report that NSSI was negatively related to peer communication [28, 32] and positively related to peer alienation [28, 33]. Similarly, a recent study by Cerutti, Zuffiano, and Spensieri [34] suggest that poor attachment may be associated with an increased difficulty for adolescents in naming and describing their own feelings, which, in turn, may increase the risk of NSSI. Yet, on the other hand, other studies have failed to observe any association between peer attachment and NSSI. For example, in a large sample of female adolescents, Lundh and colleagues [35] observed that NSSI was more strongly associated with the quality of relationships with parents than with the quality of relationships with peers. In fact, participants reporting a positive relation with their peers were found to be more vulnerable to NSSI if they had a poor relationship with their parents. A more recent study by Jiang, You, Zheng, and Lin [33] in 658 secondary school students, the authors failed to observe any association between peer communication, trust, and alienation and NSSI as well. Similarly, Yurkowski and colleagues [29] also found parental alienation to predict the probability of engaging in NSSI when controlling for dimensions of peer attachment. Based on the literature reviewed so far, the quality of relationships with parents seems to be a stronger predictor of NSSI than the quality of relationships with peers.

### Attachment and identity formation

The resolution of the identity crisis phase requires exploration of identity alternatives which can trigger confusion and ambiguity [36]. Disturbances in the development of attachment patterns can negatively impact the process of identity formation as the ability to regulate emotions may be compromised [22, 37]. There is some evidence suggesting a strong positive relation between a warm supportive relationship with parents and the formation of a coherent and mature identity in adolescence [6, 38]. Maternal attachment may be more influential in this respect [10] compared to paternal attachment. Research in adolescents from various ethnic backgrounds has indeed demonstrated that positive communication with mother was associated with the exploration of identity alternatives [38]. Additionally, the influence of parental attachment on identity formation may be stronger in females than in males [39]. A strong mother-daughter relationship has been demonstrated to be positively associated with the degree to which one identifies with an adopted identity (i.e., identity commitment, [36]). As discussed earlier, the relationship with peers can also influence the process of identity formation. In fact, some research suggests that identity formation may be influenced more by one’s relationship with one’s peers than by the quality of one’s parental attachment. For example, using a multidimensional approach to measuring attachment, Meeus and colleagues [38] demonstrated that in adolescents, greater communication and trust in peers was positively associated with exploration of identity-related options and the process of committing to one of the options. From the short review presented above, it appears that maternal and peer attachment may be relevant in the process of identity formation.

### The present study

The literature presented so far indicated that attachment, identity formation, and NSSI are likely to be interrelated. As previously mentioned, using an adolescent sample, Gandhi and colleagues [8] tested mediation models combining the above-mentioned variables using baseline data of the present study. They found that the association between the dimensions of maternal and peer attachment and NSSI may be mediated by identity synthesis and confusion. However, as their study (and most of the literature presented so far) was based on cross-sectional data, the directionality of effects among the three variables of interest was theoretically imposed. The present study is a longitudinal extension of the mediation models reported by Gandhi and colleagues [8].

In the present study, the longitudinal validity of the mediation model linking attachment, identity synthesis/confusion, and lifetime NSSI, as proposed by Gandhi and colleagues [8], was investigated using a between-person and a within-person approach. The use of longitudinal cross-lagged mediation analysis allowed us to address the issue of directionality of effects at the between-person level. More specifically, we investigated if: (a) the association between maternal and peer attachment at Time 1 (T1) and NSSI at Time 3 (T3) was mediated via identity synthesis and confusion at Time 2 (T2); or (b) engagement in NSSI at T1 predicted maternal/peer attachment at T3 through the mediating effect of identity synthesis and confusion at T2. We expected a bi-directional association between the study variables (i.e. attachment T1 → identity synthesis/confusion T2 → NSSI T3, and NSSI T1 → identity synthesis/confusion T2 → attachment T3; [8]).

## Method

### Participants and procedure

Data for the present longitudinal study were collected from students in a high school (grade 7th to 12th) located in the Dutch-speaking part of Belgium using three measurement waves. The first measurement wave was collected at the beginning of 2015 and the subsequent two waves were collected 1 year apart. Five hundred and twenty-eight, 384, and 326 students participated in the first, second, and third data collection waves, respectively. Consequently, the attrition rate at T2 was 27.3% and 39.9% at T3. On average, the number of males to females remained almost equal throughout the three waves (50.4%, 52.7%, and 54.9% females at T1, 2, and 3 respectively). The mean ages at T1, 2, and 3 were 15.0 (*SD* = 1.85 years), 15.5 (*SD* = 1.68 years), and 16.3 (*SD *= 1.65 years) respectively. To investigate if the data were missing completely at random, Little’s MCAR test was performed. The Little’s MCAR test obtained for this study’s data resulted in a Chi square estimate of 177.352, (*df *= 197; *p *= .830), which indicates that the data were likely missing completely at random.

Students were required to have written informed consent from their parents to participate in the study. Data collection was carried out during school hours. Students were provided with an envelope including assent/consent form and the questionnaires. They were requested to return the completed forms in a sealed envelope to the researchers who were present throughout the data collection process. The same procedure was used at T2 and T3. Additionally, at both T2 and T3, participants who had completed their high school education or left the school for other reasons were contacted via email and requested to complete the questionnaires online. Students were compensated with a movie ticket every time they participated in the study. To ensure confidentiality, all students were assigned a unique code number. The study was approved by the ethics committee of the Faculty of Psychology and Educational Sciences, KU Leuven (University of Leuven).

### Questionnaires

#### Non-suicidal self-injury

At T1, the lifetime prevalence of NSSI was assessed by means of a single-item question: ‘Have you ever engaged in self-injury without an intent to die?’ (answer format 0/1). At T2 and T3, 12-months prevalence of NSSI was assessed by means of the single-item question: ‘In the past 12 months, have you deliberately injured yourself without an intent to die?’ (answer format 0/1). Use of such a single-item measure is common in NSSI research [2].

#### Inventory of parent and peer attachment

Information regarding the maternal and peer attachment at T1, T2, and T3 was collected using the Inventory of parent and peer attachment (IPPS; [27]). The IPPA is a self-report questionnaire which assesses the affective and cognitive dimensions of adolescents’ relationships with their parents and peers. The present study used an abbreviated version of the questionnaire with 12 questions measuring maternal, paternal and peer related attachment. Each item is rated on a 5-point Likert scale ranging from 1 (*Never/almost never true*) to 5 (*almost always/always true*).

The IPPA scale has a three-factor structure for maternal and peer attachment [27]: (a) trust: this dimension measures the perceived degree of respect and mutual understanding in a relationship (sample item: “I like to hear the opinion of my friends/mother about things important to me”); (b) communication: this dimension measures the extent and perceived quality of spoken communication (sample item: “When I am angry about something, my friends/mother try to understand that”); and (c) alienation: this dimension assesses feelings of anger and interpersonal alienation (sample item: “It seems like my friends/mother are annoyed by me for no apparent reason”). Each factor measures a dimension of attachment. Cronbach alphas for the subscales of maternal and peer attachment at T1, T2, and T3 are given in Table 1.

**Table 1:** Cronbach’s alphas of the scales included in the study

	Time 1	Time 2	Time 3
Identity			
Synthesis	.749	.742	.794
Confusion	.671	.702	.745
Maternal attachment			
Communication	.758	.791	.799
Trust	.763	.773	.800
Alienation	.623	.674	.647
Peer attachment			
Communication	.742	.743	.797
Trust	.793	.782	.814
Alienation	.665	.678	.729

#### Identity formation

The identity subscale of Erikson Psychological Stage Inventory (EPSI; [40]) is a 12-item scale used to measure identity synthesis and confusion. Both synthesis and confusion are measured with 6 items each. Sample items for identity synthesis include “I’ve got a clear idea of what I want to be” and for identity confusion include “I don’t really know who I am.” Each item is scored on a 5-point Likert scale ranging from 1 (*Totally disagree*) to 5 (*Totally agree*). Refer to Table 1 for Cronbach alphas for the identity subscales at T1, T2, and T3.

### Analytical strategy

All the analysis was performed using Mplus (v7.4, [41]) Longitudinal measurement invariance of IPPA and EPSI was established using confirmatory factor analysis (detailed information can be accessed through Additional file 1).

**Fig. 1:**
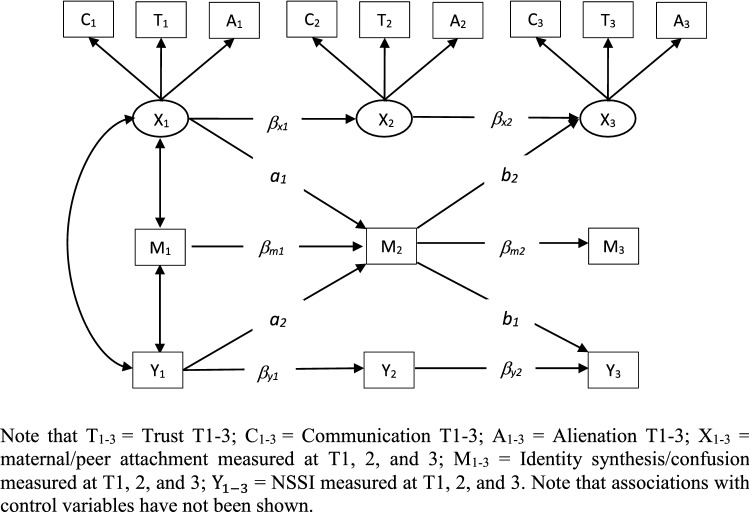
The cross-lagged mediation model tested in the present study (simplified)

The cross-lagged mediation model is shown in Fig. 1. In the cross-lagged models, coefficients *β*_*x*1_, *β*_*x*2_, *β*_*m*1_, *β*_*m*2_, *β*_*y*1_ and *β*_*y*2_ represent the stability pathways of study variables across the three measurement waves. The product of coefficients *a*_*1*_ and *b*_*1*_ measures the indirect effect from peer/maternal attachment at T1 to NSSI at T3 through identity synthesis/confusion at T2. On the other hand, the product of coefficients *a*_*2*_ and *b*_*2*_ measures the indirect effect from NSSI at T1 to peer/maternal attachment at T3 through identity synthesis/confusion at T2. Both indirect effects (*a*_1_*b_*1*_ *and a*_2_*b_*2*_) were included in the model as bidirectional associations between attachment and NSSI were expected [8]. The significance of the indirect effects was evaluated using the bootstrap procedure (5000 draws).

Although the aforementioned cross-lagged analysis approach (or its variations like random intercept cross-lagged analysis [42]) remains one of the only analytical strategies for determining the directionality of effects between variables, with this analysis, only between-person change can be modelled. However, repeated measurement of variables in a longitudinal design introduces additional within-person variability. Within-person sampling variability can bias the estimation of true variance of the person-specific mean and, in turn, bias between-person effects [43]. Therefore, in order to ensure robustness and methodological precision, an additional analysis using techniques that take into account within-person variability may be necessary. More specifically we investigated if (a) the association between the slope of maternal and peer attachment and the slope of NSSI was mediated via the slope of identity synthesis and confusion; or (b) the association between the slope of NSSI and maternal and peer attachment was mediated via the slope of identity synthesis and confusion. To investigate whether the indirect effect tested at the between-personal level was also significant at the within-person level, parallel process LGCM was used. LGCM captures the collection of individual trajectories over time in form of fixed and random effects [44]. The fixed effects are estimates of the mean intercept and mean slope. On the other hand, the random effects indicate the variance of individual values from the mean intercept and mean slope.

**Fig. 2:**
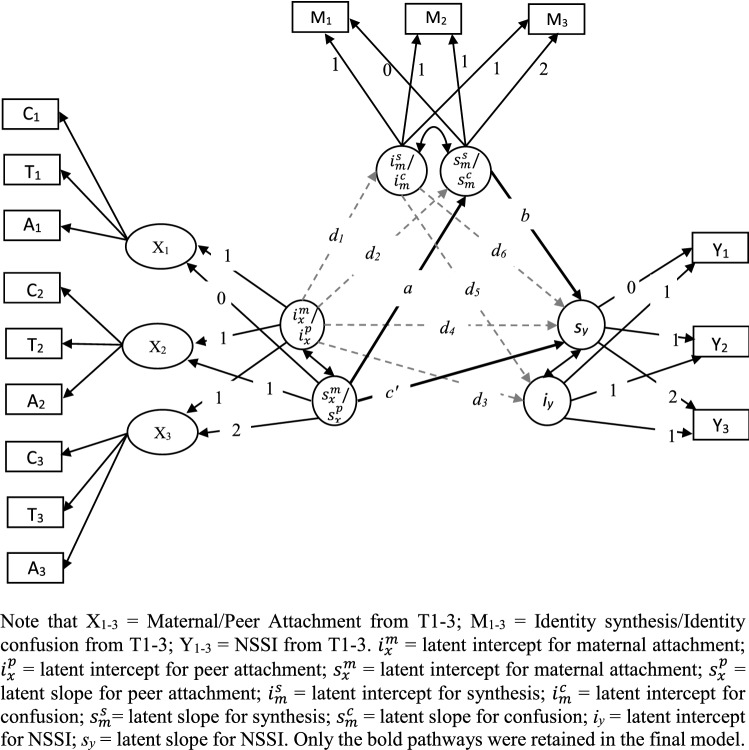
The reduced parallel process LGCM mediation models tested in the present study

To restrict the number of models being tested, only models with significant indirect effects at the between-personal level were also tested for significance at the within-person level. An example of the parallel process LGCM mediation model tested in the present work is shown in Fig. 2 (cf. [45]). Latent variables $i_{x}^{m} ,\;i_{x}^{p} ,\;i_{m}^{s} ,\;i_{m}^{c} ,\;{\text{and}}\;i_{y}$ represent the means of the intercepts and $s_{x}^{m} ,\;s_{x}^{p} ,\;s_{m}^{s} ,\;s_{m}^{c} ,\;{\text{and}}\;s_{y}$ represent the means of the slopes of the trajectories of maternal and peer attachment, identity synthesis and confusion, and NSSI. The product of the coefficients *a* and *b* represents the indirect effect of slopes of maternal and peer attachment on the slope of NSSI through the slopes of identity synthesis and confusion. To investigate indirect effects not confounded by initial differences between subjects, associations between intercepts and slopes in the variables were also added as controls (i.e., coefficients *d*_*1*_–*d*_*6*_). To make the model more parsimonious, the non-significant *d*_*1*–*6*_ pathways were removed from the mediation model. The significance of the indirect effects was again evaluated using the bootstrap procedure (5000 draws).

Given the categorical nature of the outcome variable (NSSI), weighted least square mean variance (WLSMV) with theta parameterization was used for model estimation. Age and gender were added to the model as covariates by regressing all variables of interest on these variables. Chi square value, Root Mean Square Error of Approximation (RMSEA), Comparative Fit Index (CFI), and Tucker and Lewis (TFI) were used in order to evaluate the individual model fit. As the Chi square statistic is sensitive to sample size, it was only used as a rough indicator of model fit. Models with RMSEA value of less than .08, and CFI/TFI value of more than .90 were considered to have an acceptable fit to the data [46]. Modification indices (MI) were used to handle local misfits or to add co-variances between variables to improve overall model fit. The *diff*-test procedure in M*plus* was used to compare nested models.

## Results

### Measurement invariance

Findings for the IPPA scale indicated that the partial scalar model fitted the data better than the full metric model. This indicated that the relationships of the indicators (alienation, trust, and communication) to the latent factor of (maternal and peer) attachment was equivalent through Times 1, 2 and 3. Similarly, overall analysis indicated that partial scalar measurement invariance of EPSI could be achieved over time—that is, the relationships of the indicators to the latent factors of identity synthesis and confusion were equivalent through TIMES 1, 2 and 3. For more information regarding measurement invariance of the IPPA and the EPSI scale please refer to Additional file 1.

### Cross-lagged mediation analysis

The fit indices of the four cross-lagged models are shown in Table 2. Given that the models involving maternal attachment had a good fit to the data, no specific modifications were made to these models. On the other hand, correlations between the dimensions of peer trust and peer communication within each measurement wave were required to improve the fit of the models involving peer attachment. The estimates and the indirect effects of the cross-lagged models used to investigate the directionality of effects are presented in Table 2. The indirect effect of maternal attachment at T1 on NSSI at T3 through identity synthesis and identity confusion at T2 was significant as the bias corrected 95% confidence intervals around the indirect effects did not include zero. On the other hand, the indirect effect of NSSI at T1 on maternal attachment at T3 through identity synthesis and identity confusion at T2 was not significant as the bias-corrected confidence intervals included zero. Further, based on the upper and lower limits of the bias corrected bootstrap, it can also be seen that the indirect effects of peer attachment at T1 on NSSI at T3 through identity synthesis and identity confusion at T2 were significant; whereas the indirect effects of NSSI at T1 on peer attachment at T3 through identity synthesis and identity confusion at T2 were not significant. Overall, the cross-lagged analysis indicated that the association between the variables of interest were likely to be unidirectional (i.e. from maternal/peer attachment at T1 → identity synthesis/confusion at T2 → NSSI at T3).

**Table 2:** Fit indices, standardized beta coefficients, and indirect effect with 95% bootstrap confidence intervals for cross-lagged models showed in Fig. 1

Sr. no.	Estimator	Model 1	Model 2	Model 3	Model 4
Model fits					
1	$\chi^{2} fit \left( {df} \right)$	168.54 (105)**	170.38 (104)***	258.26 (99)***	254.30 (97)***
2	RMSEA	.034	.036	.055	.055
3	CFI	.967	.961	.901	.901
4	TFI	.957	.949	.865	.863
Model coefficient estimator (S.E.)					
1	*β*_*x1*_	.88 (.04)***	.87 (.04)***	.82 (.07)***	.85 (.06)**
2	*β*_*x2*_	.79 (.05)***	.76 (.06)***	.62 (.07)***	.56 (.10)***
3	*β*_*m1*_	.45 (.07)***	.56 (.05)***	.39 (.04)**	.46 (.06)***
4	*β*_*m2*_	.74 (.05)***	.84 (.04)***	.74 (.05)***	.76 (.04)***
5	*β*_*y1*_	.89 (.08)**	.90 (.08)***	.84 (.09)***	.84 (.09)*
6	*β*_*y2*_	.60 (.11)**	.64 (.12)***	.59 (.11)***	.60 (.12)***
7	*a*_*1*_	.16 (.07)*	− .17 (.06)*	.32 (.08)***	− .40 (.07)***
8	*b*_*1*_	− .29 (.18)*	.21 (.12), *ns*	.10 (.07), *ns*	.21 (.11), *ns*
9	*a*_*2*_	− .23 (.08)*	.16 (.07)*	− .11 (.07), *ns*	.12 (.07), *ns*
10	*b*_*2*_	− .02 (.05), *ns*	− .04 (.06), *ns*	− .31 (.12)*	− .15 (.10), *ns*
Indirect effect [95% bootstrap confidence intervals]					
1	*a*_*1*_ *b*_*1*_	− .13 [− .35, − .01]	− .09 [− .24, − .002]	− .47 [− 1.01, − .13]	− .48 [− 1.03, − .04]
2	*a*_*2*_ *b*_*2*_	.00 [− .01, .01]	.00 [− .02, .00]	.00 [− .02, .00]	.00 [− .01, .00]

Model 1 = maternal attachment → synthesis → NSSI; Model 2 = maternal attachment → confusion → NSSI; Model 3 = peer attachment → synthesis → NSSI; Model 4 = peer attachment → confusion → NSSI

* *p* < .05; ** *p* < .01; *** *p* < .001

### Parallel process LGCM mediation analysis

As the association between the variables of interest was found to be unidirectional, only parallel process LGCM mediation models with maternal and peer attachment as the independent variables, identity synthesis and confusion as the mediators, and NSSI as the outcome variable were tested. The fit indices of the four-parallel process LGCM are shown in Table 3. The LGCM mediation models for peer attachment did not converge (peer attachment → identity variables → NSSI). To make the maternal LGCM models more parsimonious, age and gender were not controlled for. This had little impact on the overall model fits as almost all the associations including age and gender were not significant. Each model was further trimmed by removing non-significant pathways between the intercepts, followed by non-significant pathways between the intercepts and the slopes. Ultimately, in most LCGM mediation models, only the pathways between the slopes were retained. Table 3 indicates that indirect effects through the slopes of identity synthesis and confusion (i.e., slope of maternal attachment → slope of identity synthesis and confusion → slope of NSSI) were significant. That is, the association between the slopes of maternal attachment and the slopes of NSSI may be mediated by the slopes of identity synthesis and identity confusion. It should be noted that although pathways *b* and *c’* (see Fig. 2) were not significant in the maternal attachment models, Hayes [47] states that the overall indirect effects can still be significant, and as such, can still be interpreted.

**Table 3:** Fit indices, standardized beta coefficients, and indirect effect with 95% bootstrap confidence intervals for cross-lagged models showed in Fig. 2

Sr. no.	Estimator	Model 1	Model 2	Model 3	Model 4
Model fits					
1	$\chi^{2} fit \left( {df} \right)$	113.92 (108)*	124.68 (81)*	Models 3 and 4 did not converge	
2	RMSEA	.028	.032		
3	CFI	.981	.974		
4	TFI	.976	.966		
Model coefficient estimator (S.E.)					
1	*a*	.81 (.21)***	− .74 (.26)**		
2	*b*	− .67 (.60), *ns*	− .44 (.72), *ns*		
3	*c’*	− .45 (.63), *ns*	.63 (.65), *ns*		
Indirect effect [95% bootstrap confidence intervals]					
1	*ab*	− 7.82 [− 55.06, − 1.77]	− 1.21 [− 8.54, − .15]		

Model 1 = maternal attachment → synthesis → NSSI; Model 2 = maternal attachment → confusion → NSSI; Model 3 = peer attachment → synthesis → NSSI; Model 4 = peer attachment → confusion → NSSI

* *p* < .05; ** *p* < .01; *** *p* < .001

## Discussion

The current study integrated interpersonal (maternal and peer attachment), and intrapersonal (identity formation) processes into a single model to investigate their interplay on NSSI engagement. We investigated this interplay using a between-person (cross-lagged analytical procedure) and a more robust within-person model (parallel process LGCM analytical procedure).

Contrary to our expectation, the findings of the cross-lagged analysis indicated that the associations between attachment, identity, and NSSI were likely to be unidirectional (i.e., from attachment → identity → NSSI) and not bi-directional. Our findings support the assertion that maternal/peer attachment and identity formation can be important developmental processes that can increase vulnerability to NSSI. In fact, NSSI seems to be an outcome of a cascade of failing developmental processes. More specifically, dysfunctional attachment with either mother or peers can disrupt the process of identity formation by preventing identity synthesis and increasing identity confusion. Issues in the process of identity formation (characterised by low synthesis and higher confusion) can contribute to increased probability of engaging in NSSI.

The interplay of attachment and identity formation as vulnerability factors to NSSI remained significant (at least for maternal attachment) even when we controlled for within-person variability in the data. The use of the within-person analytical strategy of LGCM also allowed us to determine the indirect effects based on the relations between the within-individual changes in attachment, identity, and NSSI [45]. In-line with between person analysis, the within-person analysis indicated that the association between the rate of growth of maternal attachment and the rate of growth of NSSI was mediated through the rate of growth of identity synthesis and confusion. More specifically, a positive association between the growth rate of maternal attachment and the growth rate of identity synthesis. That is, improving maternal attachment may contribute to increase in identity synthesis. On the other hand, as the association between the growth rate of maternal attachment and the growth rate of identity confusion was negative, we can conclude that improving maternal attachment can deaccelerate confusion regarding the self. Unexpectedly, the association between the growth rates of maternal attachment and NSSI (*c’* in Fig. 2) and identity and NSSI (pathway *b* in Fig. 2) were found to be non-significant. In case of pathways *c’* and *b*, low power due to smaller number of students engaging in NSSI at T2 (*n *= 29) and T3 (*n *= 30) may have prevented us from reaching statistical significance.

Contextualizing our findings within the theoretical foundations of Calkins and Hill [21] and Linehan [22], we may conclude that lack of maternal attachment can hinder the emotional regulation ability of adolescents which, in turn, may have a pervasive effect on other developmental processes and identity formation in particular. In fact, Linehan suggests that emotional lability may lead to volatile behavioural reactions and cognitive inconsistencies, which may prevent formation of a stable sense of identity [22]. As suggested by Gandhi and colleagues [8] and others, a lack of a consistent sense of self may invoke strong negative affect. However, given the already hindered ability to regulate emotions, these individuals may engage in self-destructive behaviours like NSSI to deescalate the hyper-arousal state.

In contrast to the maternal attachment mediation models, LGCM models for peer attachment did not converge. The non-convergence of the LGCM mediation models involving peer attachment could be because of the very small variance of the latent slope of the peer attachment (.007) observed in these models. Small variance of random effects is known to cause convergence issues in complex LGCM models [41]. Lack of convergence of more robust longitudinal models for peer attachment does tentatively indicate that peer attachment may be a weaker predictor of NSSI. However, replication of our study with a larger sample size may be required to support this conclusion.

The findings of the current study should be interpreted in the context of the following limitations. First, the relatively small number of adolescents engaging in NSSI at T2 and T3 can lead to type 2 errors. That is, some significant effects may have been missed. Therefore, as previously noted, replications of the current findings using a larger sample size may be required. Second, although the present manuscript tested multiple models, we did not correct for multiple testing. Therefore, the possibility of increased type 1 error cannot be entirely denied. We acknowledge that using a Bonferroni corrected *p*-value would have allowed us to control for type-1 errors. However, the possibility of committing type-2 errors would have been higher as a small number of NSSI events were observed at T2 and T3. Third, the problem of missing data due to drop-outs may also be relevant, especially since we did not used partial information maximum likelihood procedure like WLSMV. Although the models tested in the current study are likely to be reliable as the WLSMV has been shown to lead to unbiased estimates even when missingness is present [48], evaluating the influence of missingness on the model estimates using sensitivity analysis is recommended [49]. Finally, in line with Gandhi and colleagues [8], we measured the association between maternal and peer attachment and NSSI. Further research should also investigate the influence of paternal attachment on NSSI.

In spite of these limitations, the current study is one of the first to assess between-person and within-person longitudinal mediational models which explored the association between developmentally relevant variables like attachment, identity, and NSSI. From a clinical perspective, our findings indicate that working with the mothers of the adolescents engaging in self-harm may be helpful in managing NSSI. NSSI has often being conceptualized as a high-cost communication behavior which adolescents use only when other low-cost behaviors have failed to elicit a response from an unresponsive environment [1]. Therefore, clinicians may consider paying specific attention to developing behavioral and emotional responsiveness of the primary care takers in families with a self-harming adolescents. Encouraging the development of healthier and more integrated identities also appears to play some preventive role. To this end, therapeutic techniques like dialectic behavior therapy-adolescents (DBT-A) have shown significant efficacy in the management of identity disturbances along with reduction in the number of NSSI episodes in adolescent [50]. Other programmes that encourage self-exploration through processes like adventure programs [51], art therapy [52], and educational means [53], may also be efficacious in helping individuals resolve issues associated with identity formation.

## Additional file

**Additional file 1: Table S1–S3.** Longitudinal measurement invariance of the Inventory of Parent and Peer Attachment (Maternal and Peer) and Identity subscale of Erikson Psychosocial Inventory.
