# Supplementary material for: Maternal and peer attachment, identity formation, and non-suicidal self-injury: a longitudinal mediation study
Source: Child Adolesc Psychiatry Ment Health. 2019 Jan 19;13:7. doi: 10.1186/s13034-019-0267-2 (PMC6339302; doi:10.1186/s13034-019-0267-2)
Supplement: Supplementary file 1 — Additional file 1: Table S1–S3. Longitudinal measurement invariance of the Inventory of Parent and Peer Attachment (Maternal and Peer) and Identity subscale of Erikson Psychosocial Inventory. [file 13034_2019_267_MOESM1_ESM.docx]

***Additional***

INDEX

| Sr. No | Content | Page no. |
| --- | --- | --- |
| 1 | Longitudinal measurement invariance:  The inventory of parent and peer attachment (IPPA): Maternal | 2 |
| 2 | Longitudinal measurement invariance:  The inventory of parent and peer attachment (IPPA): Peers | 4 |
| 3 | Longitudinal measurement invariance:  Identity subscale of Erikson Psychosocial Inventory (EPSI) | 6 |

*1. Longitudinal measurement invariance IPPA: Maternal attachment*

Table S1. Comparison of configural, metric, scalar and partial scalar models for longitudinal measurement invariance two factor solution for IPPA (Mother).

| Sr.  no | Model  (Three-factor) | $\chi^{2}$ | *df* | *p* | $\chi^{2}$ test | | | CFI | TFI | RMSEA |
| --- | --- | --- | --- | --- | --- | --- | --- | --- | --- | --- |
|  |  |  |  |  | $\Delta\chi^{2}$ | $\Delta df$ | Critical |  |  |  |
| 1 | Configural | 6.663 | 15 | .312 |  |  |  | 1.00 | 1.00 | .000 |
| 2 | Metric | 8.584 | 19 | .979 | 1.92 | 4 | 9.49 | 1.00 | 1.00 | .000 |
| 3 | Scalar | 11.965 | 25 | .987 | 3.29 | 6 | 12.59 | 1.00 | 1.00 | .00 |

Like the two-factor solution for peer attachment, the two-factor solution for maternal attachment suggested by Gandhi and colleagues (2015) and Johnson and colleagues (2003) also did not converge in spite of making multiple adjustments to the model. Therefore, the metric and scalar models for the two-factor solution were not tested. Table 6. shows the fit indices and adjusted $\chi^{2}$ test for model comparisons of the models used to investigate the longitudinal measurement invariance of three-factor solution of the maternal attachment sub-scale of the IPPA. The configural model (i.e., model 1) had a good fit and the standardised factor loadings ranged from -.700 to .921. To facilitate convergence, residual correlations between the communication factor measured across time were left uncorrelated. No further modifications were made to the model because it had an acceptable fit. The metric model also had a good fit (see Table 1.) and from the results of the adjusted $\chi^{2}$ test, it was clear that fit of the metric model was not worse than the configural model. Finally, even the full scalar model had an adequate fit to the data and a non-significant adjusted $\chi^{2}$ test indicated that the model fit was not significantly worse than the metric model. Overall, the analyses showed that the relationships of the indicators (alienation, trust, and communication) to the latent factor of maternal attachment were equivalent through Times 1, 2 and 3. The factor loadings of the scalar model for maternal attachment ranged from -.704 to .916.

*2. Longitudinal measurement invariance IPPA: Peer attachment*

Table S2. Comparison of configural, metric, scalar and partial scalar models for longitudinal measurement invariance two factor solution for IPPA (Peer).

| Sr.  no | Model  (Three-factor) | $\chi^{2}$ | *df* | *p* | $\chi^{2}$ test | | | CFI | TFI | RMSEA |
| --- | --- | --- | --- | --- | --- | --- | --- | --- | --- | --- |
|  |  |  |  |  | $\Delta\chi^{2}$ | $\Delta df$ | Critical |  |  |  |
| 1 | Configural | 32.895 | 18 | .017 |  |  |  | .989 | .977 | .040 |
| 2 | Metric | 36.340 | 22 | .028 | 3.68 | 4 | 9.49 | .987 | .978 | .038 |
| 3 | Scalar | 68.243 | 28 | <.001 | 29.24 | 6 | 12.59 | .969 | .960 | .052 |
| 4 | Partial Scalar* | 39.67 | 26 | .042 | 3.765 | 4 | 9.49 | .989 | .985 | .032 |

*Difftest in comparison to model (2)

The configural measurement invariance model for two-factor structure of IPPA suggested by Gandhi and colleagues (2015) and Johnson and colleagues (2003) did not converge in spite of making multiple adjustments to the model. As the confirgural model did not converge, metric and scalar models for the two-factor solution were not tested. Table 7 shows the fit indices and adjusted $\chi^{2}$ test for model comparisons of the models used to investigate the longitudinal measurement invariance of the peer attachment sub-scale of the IPPA scale. The configural model (i.e., model 1) had a good fit and the standardised factor loadings ranged from -.524 to .977. No modifications were made to the model because it had a good fit and this model was used as the baseline configural model. Note that, in order to facilitate convergence, residual correlations between the communication factor measured across time were left uncorrelated.

The metric model also had a good fit (see Table 2) and from the results of the adjusted $\chi^{2}$ test, it was clear that fit of the metric model did not have a worse fit than the configural model. Again, no changes were made to the model as the model fit of the metric model was already good. Finally, although the full scalar model had an adequate fit to the data, a significant adjusted $\chi^{2}$ test indicated that the model fit was worse than the full metric model. Modification indices were inspected to identify causes of local misfit in the full scalar model. Modification indices indicated that the fit would improve if the intercepts of the communication factor was freely estimated. A partial scalar model with freely estimated intercept of communication factor was tested. The fit of the partial scalar model improved substantially and the model did not have significantly worse fit than the metric model. Overall, the analyses showed that the relationships of the indicators (alienation, trust, and communication) to the latent factor of attachment were equivalent through Times 1, 2 and 3. The factor loadings in the partial scalar model ranged from -.553 to .972.

*3. Longitudinal measurement invariance: EPSI*

Table S3. Comparison of configural, metric, scalar and partial scalar models for longitudinal measurement invariance two factor solution for EPSI.

| Sr.  no | Model | $\chi^{2}$ | *df* | *p* | Difftest (MPlus procedure) | | | CFI | TFI | RMSEA |
| --- | --- | --- | --- | --- | --- | --- | --- | --- | --- | --- |
|  |  |  |  |  | $\Delta\chi^{2}$ | $\Delta df$ | *p* |  |  |  |
| 0 | Configural | 905.501 | 543 | <.001 |  |  |  | .965 | .959 | .036 |
| 2 | Metric | 887.204 | 587 | <.001 | 47.604 | 44 | .328 | .971 | .969 | .031 |
| 3 | Scalar | 1298.573 | 658 | <.001 | 615.189 | 71 | <.001 | .938 | .941 | .043 |
| 4 | Partial Scalar* | 925.792 | 656 | <.001 | 81.232 | 69 | .149 | .971 | .972 | .029 |

*Difftest in comparison to model (2)

Table 5. shows the fit indices and difftest procedure for model comparisons tested to investigate the longitudinal measurement invariance of the EPSI scale. In the first configural model (i.e., model zero), the factor structure suggested by Schwartz and colleagues (2009) was followed, i.e., items 2, 4, 5, 6, 8, and 9 were loaded on synthesis and items 1, 3, 7, 10, 11, and 12 were allowed to load on the confusion subscale. From table 3, it is clear that this model had an acceptable fit. Examination of the factor loading however demonstrated that factors eight (factor loading ranged from .267 to .326) and 11 (factor loadings ranged from .207 to .284) less than .40 – a commonly accepted cut off value for factor loadings. Given that small number of questions in both identity synthesis and confusion subscales, items with factor loading lesser than .40 were not removed. The resulting configural model (i.e., model 1) had a good fit and no further modification were done to the model. The standardised factor loadings of the remaining items in the second configural model ranged from .267 to .873.

The metric model also had a good fit (see Table 3.) and from the results of the diff test, it was clear that fit of the metric model did not have a worse fit as compared to the configural model. Fewer modification indices were available at this step, yet no changes were made to the model as the model fit of the metric model was already good. Finally, although the full scalar model had an adequate fit to the data, a significant difftest indicated that the model fit was worse than the full metric model measured in the previous step. Modification indices were inspected to identify causes of local misfit in the full scalar model. Modification indices indicated that the fit would improve if the second threshold of item 3 at time 3 was estimated freely. The second thresholds of all item three were freely estimated as these thresholds also had high EPC. The fit of the partial scalar model improved substantially and the model did not have significantly worse fit than the metric model. In conclusion, these analyses showed that partial measurement invariance of EPSI could be achieved over time – that is, the relationships of the indicators to the latent factor of identity synthesis and confusion were equivalent through Times 1, 2 and 3.
